# Master of Science (MSc) Program in Radiation Biology: An Interdepartmental Course Bridging the Gap between Radiation-Related Preclinical and Clinical Disciplines to Prepare Next-Generation Medical Scientists

**DOI:** 10.3389/fonc.2017.00226

**Published:** 2017-09-22

**Authors:** Stephanie E. Combs, Carmen Kessel, Jan J. Wilkens, Gabriele Multhoff, Thomas E. Schmid, Peter Vaupel, Klaus-Rüdiger Trott, Pascal Berberat, Michael J. Atkinson

**Affiliations:** ^1^Department of Radiation Oncology, Klinikum rechts der Isar, Technical University of Munich (TUM), München, Germany; ^2^Department of Radiation Sciences (DRS), Institute of Innovative Radiotherapy (iRT), Helmholtz Zentrum München (HMGU), Neuherberg, Germany; ^3^Deutsches Konsortium für Translationale Krebsforschung (dktk), Partner Site Munich, Munich, Germany; ^4^Physics Department, Technical University of Munich (TUM), Garching, Germany; ^5^TUM Medical Education Center, School of Medicine, Technical University of Munich (TUM), München, Germany; ^6^Department of Radiation Sciences (DRS), Institute of Radiation Biology (ISB), Helmholtz Zentrum München (HMGU), Neuherberg, Germany; ^7^Radiation Biology, Technical University of Munich (TUM), München, Germany

**Keywords:** molecular biology, radiation protection, physics, immunology, medical informatics, clinical radiation sciences, education, teaching

## Abstract

Radiation biology is a highly interdisciplinary field at the interface of biology, physics, and medicine. It is characterized by rapid advances in biological and technical knowledge. The potential for using these advances to optimize medical care, radiation protection, and related fields can be exploited only with complementary activities to support the education of young academics. A small number of academic institutions have committed resources into radiation-related courses and curricula; however, few offer a comprehensive interdepartmental research and training program. At the Technical University of Munich (TUM), a full Master of Science (MSc) course in radiation biology has been established. This article describes the TUM MSc radiation biology program, discusses the scope of the field, the teaching goals, and the interdisciplinary curriculum. Detailed information on the full MSc program can be found continuously updated at www.radonc.med.tum.de/masterradiationbiology.

## Introduction: What is Radiation Biology?

“Radiation biology” is an emerging and strongly interdisciplinary field with significant contributions from a number of preclinical and clinical disciplines. In the early 1900s, radiation biologists focused on purely descriptive analyses of radiation effects on living tissue, including tumor cells and normal tissue. This led to the discovery and exploitation of such basic phenomena as dose, fractionation, hypoxia, tissue weighting, and radiation quality (1). Other disciplines focused on physical and chemical aspects of radiation and its effects on cells and tissues (2). Only in the latter half of the 20th century, did the challenges associated with understanding radiation actions prompt interdisciplinary studies from the fields of physics, biology, molecular biology, immunology, medical informatics, and several clinical disciplines including radiation oncology, radiology and nuclear medicine. Over the years, all these subtopics have developed into large subdisciplines. Unfortunately, in spite of the common core interest, no comprehensive educational program exists that can teach all aspects related to radiation biology in a single curriculum.

Historic events, such as the bombings of Hiroshima and Nagasaki, and major scientific achievements led to the awareness of the severe health effects of radiation (3–5). This prompted the search for treatment of acute radiation syndrome and for cancer treatment options sparing normal tissues. Recently the ecological consequences of radioactive contamination have become a major public concern. The knowledge of wanted and unwanted effects of radiation has undoubtedly evolved to an even greater necessity since the positive effects of radiation can be used beneficially in healthcare and daily normal life. However, increasing cure rates produce long-term survivors at risk for chronic radiation-related health effects.

Thus, the necessity to train young researchers, clinicians, and clinician scientists in all aspects of radiation biology is well recognized. Only recently, a workshop was put together at the 60th Annual Meeting of the Radiation Research Society in 2014 with the focus “Education and Training Needs in the Radiation Sciences: Problems and Potential Solutions.” Recommendations included focused education structures for young academics as well as continuing education venues (6).

Until 2012, a European consortium offered a 1-year Master of Science (MSc) degree course “Radiation Biology” at University Colleges of London (UCL), United Kingdom. The absence of a single faculty able to teach all aspects of the topic necessitated the transfer of the students to five different centers in Europe. The decision of the UK government to introduce high course fees for students led to the closure of this course and its eventual transfer to the Technical University of Munich (TUM). At the University of Oxford, United Kingdom, a 1-year full-time MSc course is being taught comprising theoretical basics, emerging areas of fundamental biology for oncology and its treatment by radiotherapy, and a 6-month high-quality basic and clinically applied research project. Other courses offer lectures on radiation and its effects in a mixed approach, such as in the MSc Radiation and Environmental Protection at the University of Surrey, UK, or various dedicated medical physics-oriented courses, e.g., at the Technical University Dresden and Heidelberg University, Germany. Most of these courses are set up as postgraduated programs offered to degree holders in the field of medicine, physics, chemistry, biology, or other related disciplines.

The MSc Radiation Biology at TUM was set up based on the long-standing experience of the UCL course. However, the teaching concept was revised as a 2-year course in Munich that is compliant with the Bologna criteria. Unlike at UCL, the Technical University of Munich (TUM) already has expertise in most topics of the MSc program, most importantly in the clinical disciplines related to radiation biology, which are represented by a high level of expertise. Within the Munich metropolitan area, several other institutions have radiation-related experts, including the research reactor Forschungsneutronenquelle Heinz Maier-Leibnitz at TUM, the Department of Radiation Sciences (DRS) at the Helmholtz Zentrum München (HMGU), and the Bundeswehr Institute of Radiobiology as well as the Bundesamt für Strahlenschutz (BfS). Thus, it was possible to establish a joint curriculum teaching all aspects related to radiation biology, bridging the gap between preclinical and clinical fields, between theoretical and practical research, and between different faculties within TUM and within different Universities in Munich.

## Radiation Biology: A European Perspective

There is a long tradition of radiation biology in Europe. Strong societal networks including the European Society for Radiation Oncology (ESTRO) and the European Radiation Research Society (formerly the European Society of Radiation Biology) already exist to foster research collaborations, data exchange, and lively discussion of novel research as well as educational programs. At regular meetings, training courses and specific basic lectures for young scientist are being held. ESTRO offers courses on radiation biology, molecular radiation biology, and medical physics or specific clinical topics in radiation oncology on a regular basis. Teaching staff is recruited from European and International institutes, generally university based. Specific undergraduate or graduate courses offering training as a broad-based curriculum exist only on a national level, however, in low numbers. Within EURATOM, education and training activities are an integral part of all multinational projects in FP7 and H2020. These include training courses and educational meetings.

## Radiation Biology: Involved Disciplines for the Development of an Interdepartmental Program

**Figure 1:**
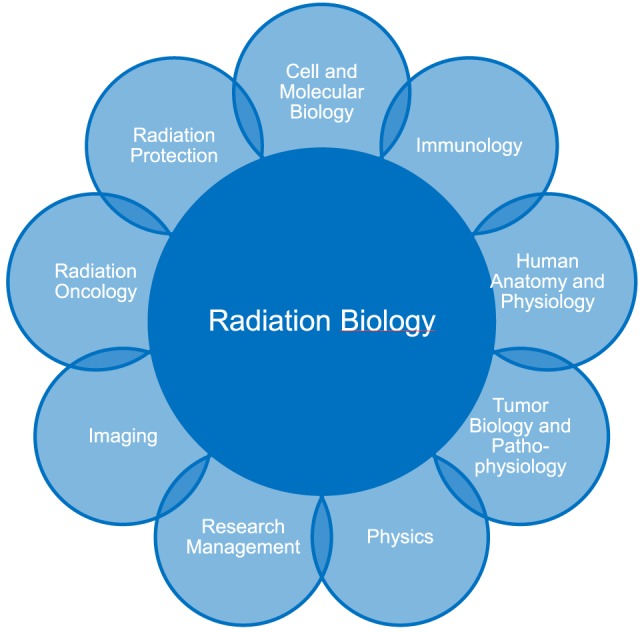
The MSc Radiation Biology was designed as an interdepartmental course including all relevant and related disciplines to the field.

In preparation of the MSc course in radiation biology, we analyzed all areas of research and teaching related to radiation biology (see interaction on Figure 1). Moreover, we identified structural possibilities and collected information about comparable interdisciplinary teaching structures worldwide (7–11). At TUM, a number of faculties, institutes, and departments have research activities related to effects of radiation on normal and diseased tissue. Several groups focus on the impact of radiation on tumors, interactions of the immune system with radiation, or physics research relating to radiation oncology and diagnostic radiology and the characterization of dose and dose calculations. Well connected within the university hospital Klinikum rechts der Isar lie the related clinical disciplines for imaging and the knowledge about the treatment of patients, as well as information technology (IT) and data management groups working on radiation oncology, radiation documentation, big data management, and the evaluation of complex responses in terms of bioinformatics and radiomics. Thus all aspects relevant to an MSc Radiation Biology are available. Since no existing program, neither on the level of graduate training nor on the level of postgraduate training, provided a comprehensive compilation of these aspects, the goal is to develop a global interdepartmental course program incorporating all relevant aspects and build on the strength of the research fields at TUM. Due to the strong interactions and double affiliations between TUM and the Department of Radiation Sciences (DRS) at the Helmholtz Zentrum München (HMGU), as well as due to the distinct expertise especially in radiation emergencies of the Bundeswehr Institute of Radiobiology, and the Bundesamt für Strahlenschutz (BfS) these institutions were included into the curriculum.

### Faculty

The MSc Radiation Biology faculty is spread across several departments at TUM. This includes topics from pure radiation biology research groups, physics, biology, physiology, immunology, radiology, radiation oncology, nuclear medicine, and pathology. Teachers are experienced university professors and researchers in their discipline. At TUM, specialized educational training is required for all tenured scientists, thus knowledge in most recent training and teaching methods including problem-oriented learning, journal clubs, interactive teaching, IT-based and online learning possibilities as well as classical lectures with interactive elements are included into the curriculum.

### Students

There is a widespread spectrum of students enrolled in the MSc course. The prerequisite being a bachelor degree in fields of life sciences such as biology, medicine, physics, molecular biology, or other related subjects. Study language is English; therefore, knowledge of the English language is necessary and must be proven upon application. The professional qualification of the students reflects the interests and topics related to radiation biology accordingly and underlines the necessity of providing a comprehensive program in this field. The interdepartmental curriculum of the MSc Radiation Biology was designed to provide a comprehensive education in all aspects relevant to the field. Students can apply according to the TUM criteria for MSc courses. In addition, we schedule interviews with the applicants to understand their motivation and to judge their qualification as well as language capacities. This selection process has been set up to select students effectively aiming at a small but high-level MSc course.

## Development of an Interdepartmental Program

Due to the interdisciplinary nature of the program, we had to decide where to affiliate such an MSc course within the TUM. Since radiation therapy, radiology, and nuclear medicine are core disciplines in medicine, it was logical to associate the course in the TUM School of Medicine. However, key expertise in biology and molecular biology is associated with the TUM School of Life Sciences. At TUM, we have the unique setting of a specialized Institute of Radiation Biology, linked to the school of medicine and the school of life sciences. In physics, a parallel situation is found: The Chair of Biomedical Physics and the Professor of Medical Radiation Physics are associated with the Physics Department as well as the School of Medicine. All other relevant disciplines including medical informatics and immunology are associated with the School of Medicine. No individual department of subspecialty would have been able to provide the comprehensive knowledge bundled in the new curriculum. Therefore, the new MSc course radiation biology represents modern cross-departmental thinking, which is necessary to synthesize all relevant strengths in the field.

## TUM Radiation Biology Curriculum

The curriculum consists of 15 modules of which the final module is the Master’s Thesis. The curriculum reflects the broad nature of the topic “Radiation Biology” as well as the special needs and demands by young academics in the field (12–15). Generally, it is understood that it is impossible for the students to learn the full complexity of each component field. Therefore, we considered it essential to extract all relevant topics and information from each discipline and to carve out the most significant features indispensable for radiation biology.

To acquire the necessary knowledge in practical lab work or clinical applications of radiation physics, dosimetry, or radiation oncology, two 6-week long research internships in either a laboratory or in a research group with focus on medical physics, clinical research, or radiation protection are obligatory during the course of the 2-year curriculum. Typically, two to four modules consisting of lectures, practical work, and exercises are offered per semester; lectures are recorded and offered *via* an e-learning environment (Moodle) for after hours study and recapitulation. As the background of the students is heterogeneous, specific tutorials are offered as a complementary source of information besides the classical lectures. With the Master’s Thesis, where students can pick their own research topic from various projects offered by the participating departments and institutions, they complete their studies and prove their scientific skills.

### Human Anatomy and Physiology

After completion of the MSc course, the students will have a thorough comprehension of human anatomy including histological features. Knowledge of the main cells types and structures found within cells and tissues. Physiology is a key element necessary to understand the clinical effects of radiation on normal and diseases tissue. Therefore, the physiological functioning of each organ is taught in tight conjunctions of organ pathology in terms of histopathological changes as well as clinical symptoms. Thus, the students will be able to understand the effects of diseases on organ function and organ structure, especially the results of radiation exposure to normal tissue.

### Tumor Biology and Tumor Pathophysiology

Besides pure anatomy and physiology, a central expertise within the MSc course is knowledge of tumor biology and pathophysiology in the teacher roster. Compared to other radiation biology courses, this is one of the unique selling points of the present MSc course. Thus, by attending the first modules of the MSc program, the students will get to know all specifics of tumor biology and the connected pathophysiology. This is important to further understand any clinical implications and the radiation-induced changes in the tumor as well as the relevant underlying mechanisms.

### Cell and Molecular Biology

The students will have a thorough comprehension of the cells and their organelles, the main molecules, and their function: DNA, RNA, metabolites, and other. Curricular elements consist of the molecular organization and functioning of cells (e.g., transcription, translation, splicing, and mutations) including extensive knowledge of damage repair mechanisms especially when there is relevant radiation damage. Finally, the students will learn how to unravel molecular and genomic information, including gene sequencing, transcriptomics, and other. The broad basis on cell and molecular biology and genetics will serve them as an important future platform to analyze and understand novel data, to describe relevant findings in the field, and to conduct research and studies unraveling novel insights.

### Immunology

The immune system is not only an armamentarium to defeat infections and other diseases but also a structure closely involved in damage to normal tissue, damage repair, and tumor growth and oncological strategies. Today, it is known that immune cells are strongly involved in metastases, tumor growth, and other aspects of tumor biology; many cancer patients additionally demonstrate immune deficits making their body prone to cancer spread dissemination. Radiation itself has a strong impact on the immune system, and radiation effects can stimulate and enhance immunogenic mechanisms. Therefore, the students will be educated starting from basic immunology, relevance of the immune system in the context of radiation biology, radiation effects on components of the immune systems, and novel strategies on how to combine immunotherapy and radiation therapy in various clinical situations.

### Radiation Physics

Physical principles are an indispensable knowledge in all fields of radiation; they are necessary to calculate radiation protection necessities, to determine doses in the environments and in medical applications, to explain the effects of radiation on tissue, and to describe and understand technical devices in the radiation field. Modern dosimetry is essential to determine doses and develop novel applications. Thus, the module radiation physics provides basic knowledge of physical principles and advanced methods and applications. The students will be able to understand and explain all relevant items in the field. They will also be able to reflect the physical principles in imaging and therapy and can point out the differential indications of each method. Moreover, they will get to know research strategies in the field of physics including data generation, presentation, and communication.

### Radiation Protection

Calculating dose in a clinical setting, environmental exposure, and nuclear accidents is highly relevant and a key component in the governmental mandate of radiation biologist. The students are educated about the relevant historical events and the data generated from these incidents, including countermeasures, when necessary. They will learn tolerance doses and the disease spectrum related to radiation exposure, including optimal treatment during the different phases. Second, they will be taught high- and low-dose radiation effects. Dose and shielding calculations will lead to strategies of radiation protection in different clinical and non-clinical scenarios.

### Radiation Oncology, Radiology, and Nuclear Medicine

In the central clinical disciplines, radiation oncology, radiology, and nuclear medicine, all information from the basic science modules will converge into clinical applications. The students will learn the differences of each discipline, the area of work, and the relevant exposures related to the different examinations and treatment schemes. The use of the ALARA principle (“as low as reasonably possible”) will be explained in the clinical context, and modern high-precision conformal treatment techniques will be taught, including stereotactic radiotherapy and particle therapy. In conjunction with the modules, cell biology, molecular biology, and immunology, a main goal is to elucidate the principles of individualized treatment concepts in personalized medicine.

### Research Management

The students will learn about data collection and analysis, basic statistical approaches to data evaluation, as well as different database structures for data management. The students will be able to perform structured searches on scientific data. To analyze large data volumes, efficient search mechanisms, sorting strategies, and data mining techniques are relevant. Structured data reporting either in written documentation such as scientific papers, white papers, or other is essential, and project management from data generation, data sorting, and data analysis including reporting will be key aspects in this module.

## Conclusion

Our MSc Radiation Biology program has been developed to educate young academics from many fields of medicine and life sciences in all relevant areas of radiation biology. The goal is to educate postgraduates competent in a broad range of subjects, techniques and immunological aspects, biology basics, as well as advanced molecular insights, all related to the central topic radiation biology. The alumni should have a clear understanding of all beneficial and detrimental effects of radiation. We expect our students to be competent in all areas related to radiation biology; however, they can choose different modules for their own focal point, e.g., radiation protection, molecular biology, or clinical and experimental radiation oncology. Depending on the chosen focus within the MSc, we expect them to be experts in the field with a strong ability to analyze data independently; advise politicians, companies, or others seeking information in this field; design research or study areas related to the field; or pursue a career as a medical or clinician scientist (16).

At the Technical University of Munich (TUM), the graduates from MSc in radiation biology may look forward to rewarding career options both in and outside of academia and industry, in many sectors of biomedical sciences, governmental organizations, or private companies.

## Author Contributions

SC wrote the manuscript and proposed the initial concept. CK advised and edited this manuscript. JW, GM, TS, PV, K-RT, PB, and MA corrected the manuscript. All authors approved the manuscript.

## Conflict of Interest Statement

The authors declare that the research was conducted in the absence of any commercial or financial relationships that could be construed as a potential conflict of interest.

## References

[1] Kaplan HS Historic milestones in radiobiology and radiation therapy. Semin Oncol (1979) 6(4):479–89. 119321

[2] Zherbin EA [Role of radiobiology in the development of radiotherapy]. Med Radiol (Mosk) (1982) 27(5):48–56. 7047971

[3] Hall EJ. The contribution of the physical sciences to the development of radiation therapy. J Surg Oncol (1983) 24(4):248–57. 10.1002/jso.2930240403 6418975

[4] Grant EJ Ozasa K Ban N de Gonzalez AB Cologne J Cullings HM A report from the 2013 international symposium: the evaluation of the effects of low-dose radiation exposure in the life span study of atomic bomb survivors and other similar studies. Health Phys (2015) 108(5):551–6. 10.1097/HP.0000000000000262 25811153

[5] Grant EJ Ozasa K Preston DL Suyama A Shimizu Y Sakata R Effects of radiation and lifestyle factors on risks of urothelial carcinoma in the life span study of atomic bomb survivors. Radiat Res (2012) 178(1):86–98. 10.1667/RR2841.1 22631857

[6] Dynlacht JR Zeman EM Held KD Deye J Vikram B Joiner MC. Education and training needs in the radiation sciences: problems and potential solutions. Radiat Res (2015) 184(5):449–55. 10.1667/RR14199.1 26479274

[7] Johnson SB Friedman RA. Bridging the gap between biological and clinical informatics in a graduate training program. J Biomed Inform (2007) 40(1):59–66. 10.1016/j.jbi.2006.02.011 16616697

[8] Gerstein M Greenbaum D Cheung K Miller PL. An interdepartmental Ph.D. program in computational biology and bioinformatics: the Yale perspective. J Biomed Inform (2007) 40(1):73–9. 10.1016/j.jbi.2006.02.008 16650809

[9] Dorsey AD Clements K Garrie RL Houser SH Berner ES. Bridging the gap: a collaborative approach to health information management and informatics education. Appl Clin Inform (2015) 6(2):211–23. 10.4338/ACI-2014-09-RA-0083 26171071 PMC4493326

[10] Chastonay P Zesiger V Moretti R Cremaschini M Bailey R Wheeler E A public health e-learning master’s programme with a focus on health workforce development targeting francophone Africa: the University of Geneva experience. Hum Resour Health (2015) 13:68. 10.1186/s12960-015-0065-8 26268723 PMC4535289

[11] Lamba S Strang A Edelman D Navedo D Soto-Greene ML Guarino AJ. Promoting interprofessionalism: initial evaluation of a Master of Science in Health Professions Education Degree Program. Adv Med Educ Pract (2016) 7:51–5. 10.2147/AMEP.S97482 26917985 PMC4751893

[12] Lopez Guerra JL Isa N Kim MM Bourgier C Marsiglia H. New perspectives in radiation oncology: young radiation oncologist point of view and challenges. Rep Pract Oncol Radiother (2012) 17(5):251–4. 10.1016/j.rpor.2012.07.002 24669303 PMC3885889

[13] Urbanski B. The future of radiation oncology: considerations of young medical doctor. Rep Pract Oncol Radiother (2012) 17(5):288–93. 10.1016/j.rpor.2012.09.002 24669310 PMC3885896

[14] Zietman A. The future of radiation oncology: the evolution, diversification, and survival of the specialty. Semin Radiat Oncol (2008) 18(3):207–13. 10.1016/j.semradonc.2008.01.009 18513631

[15] Olsson M Persson M Kaila P Wikmar LN Bostrom C. Students’ expectations when entering an interprofessional master’s degree program for health professionals: a qualitative study. J Allied Health (2013) 42(1):3–9. 23471279

[16] Smith BD Haffty BG Wilson LD Smith GL Patel AN Buchholz TA. The future of radiation oncology in the United States from 2010 to 2020: will supply keep pace with demand? J Clin Oncol (2010) 28(35):5160–5. 10.1200/JCO.2010.31.2520 20956628

